# Health Disparities in the Use of Primary Cesarean Delivery among Asian American Women

**DOI:** 10.3390/ijerph20196860

**Published:** 2023-09-29

**Authors:** Yuqing Zhang, Lisa Heelan-Fancher, Suzanne Leveille, Ling Shi

**Affiliations:** 1 College of Nursing, University of Cincinnati, Cincinnati, OH 45040, USA; 2 Robert and Donna Manning College of Nursing and Health Sciences, University of Massachusetts Boston, Boston, MA 02125, USA; lisa.heelan-fancher@umb.edu (L.H.-F.); suzanne.leveille@umb.edu (S.L.); ling.shi@umb.edu (L.S.)

**Keywords:** birth registry, Asian health disparities, primary cesarean delivery

## Abstract

This study examined the health disparities in primary cesarean delivery (PCD) use among Asian American (AA) women and within AA subgroups. We examined 22 years of birth registry data from one diverse northeastern state in the United States, including singleton vertex live births between 24 and 44 weeks of gestation without congenital abnormalities. Multivariate logistic regression was used to test the association between PCD and race and ethnicity groups adjusting for maternal demographic and health behaviors, infant gender and birth weight, gestational age, initiation of prenatal care, and other risk factors. Among the eligible sample, 8.3% were AA. AAs had the highest rate of PCD (18%) among all racial and ethnic groups. However, extensive heterogeneity was found among the AA subgroups. After controlling for confounding variables, compared to non-Hispanic White women, Filipino, Asian Indian, and Other Asian subgroups had a higher risk for PCD (Adj OR = 1.40, 1.37, and 1.21, *p* < 0.001), while Japanese, Chinese, and Korean had a lower risk (Adj OR = 0.57, 0.83, and 0.90, *p* < 0.001), and Vietnamese had no significant difference in PCD use. Although AA as a single racial and ethnic group had higher prevalence of PCD, more studies are warrantied to address the disproportional distribution of health disparities in PCD use within AA subgroups.

## 1. Introduction

One in three infants was born via cesarean delivery (CD) in the United States (US) in 2020 [1]. This is alarming because the World Health Organization [2] recognizes that, based on the evidence, CD rates at the population level higher than 15% are not associated with a reduction in maternal and newborn mortality [3,4]. In fact, women in the US who deliver via CD are three times more likely to die following childbirth compared to women who have had a vaginal birth [5]. Being born via CD also puts the infant at increased risk for respiratory distress and lower rates of breastfeeding [6]. Additionally, it takes a woman a longer time to recover from childbirth after a CD and the costs associated with a CD are 75% higher compared to a vaginal birth [7]. The high CD rate in the US is driven by the increase in primary CD (PCD) and the dramatic decrease in the use of vaginal birth after CD (VBAC) [5]. Therefore, professional organizations such as the American College of Obstetricians and Gynecologists (ACOG) have called for efforts to decrease the PCD rate [5].

Notably, there are significant and persistent racial disparities in CD rates among pregnant women in the US. For example, in 2020, Asian Americans (AA) had the second highest rate of CD (33%) in the US [1]. This is of concern because the Asian population doubled from 11 million persons in 2000 to 22.4 million in 2019. The Asian population are the fastest growing racial group in the US and are expected to be the largest immigrant group in the US by 2060 [8]. However, AAs are the least studied racial and ethnic group and have been overlooked as a population that experiences health disparities [9,10].

Although several studies have examined racial and ethnic disparities in CD/PCD use among US women [11,12,13,14,15,16,17,18,19,20], AA women were often not included in their analyses [15,18,20], or if included, they were examined as a single racial and ethnic group [11,12,13,16,17,19]. Viewing AA women from a homogeneous lens does not take into account the differences between and among Asian women and thus may be a contributing factor toward the inconsistent findings reported in previous studies as regards to PCD/CD use [11,12,13,16,17,19]. Previously, Edmonds and her colleagues examined PCD use among subgroups of AA women using birth registry data but only included nulliparous women in the sample and did not consider that PCD could have occurred among multiparous women [14]. Although the likelihood of having a PCD is higher among nulliparous women compared to multiparous women, not including multiparous women in a study using birth registry data could have potentially underestimated the use of PCD. Another limitation of this previous study was the lack of adjustment for obstetric and health risk factors such as diabetes, hypertension, induction, continuous electronic fetal monitoring, or congenital infant anomalies. In a different study, Carlson and colleagues speculated that race-related differences in fetal intolerance of labor and the use of induction might have contributed to the apparent disparities in CD/PCD prevalence in US women [15]. Additionally, the labor practice of using continuous fetal monitoring (CEFM) is an independent risk factor for the increased use of PCD [21]. Therefore, it is important to understand how race/ethnicity might influence CD/PCD use regardless of other risk factors.

The gaps in previous studies made it imperative to examine the use of PCD among AA women, especially within AA subgroups, and to control for obstetric and health risk factors. We hypothesized that the use of PCD is disproportionally and independently associated with differences based on AA ethnic subgroups even after controlling for obstetric and health risk factors. The aims of this study were to examine the prevalence of PCD use in AA women in a highly populated Northeastern state and to determine if there are differences in the prevalence of PCD among the different AA subgroups, specifically, Asian Indian, Chinese, Filipino, Japanese, Korean, and Vietnamese.

## 2. Materials and Methods

### 2.1. Participants

A secondary data analysis was performed to examine the birth registry data for the years 1992 to 2014 from one diverse populous state in the northeastern US. This study was approved by the institutional review boards (IRB) of the University of Massachusetts Boston and the respective state. The study sample included singleton vertex live births between 24 and 44 weeks of gestation in a hospital or a birth center. Excluded from our sample were mothers who self-reported as multiracial or ‘other’ race, infants with congenital abnormalities, births that occurred out of state, and births with missing items important to our data analysis such as gestational age or race and ethnicity.

### 2.2. Measures

The sociodemographic characteristics of the mothers included age (≤20, 21–34, ≥35), race and ethnicity (non-Hispanic [NH] White, NH-Black, AA, and Hispanic), marital status (married, not married), and education (less than high school, high school completed, attended college). Childbirth outcomes included in the analysis were PCD (Yes/No). Other covariates included in the analysis were the mothers’ self-reported health behaviors of drinking alcohol and use of tobacco, the infants’ gender and birthweight, the initiation of prenatal care, nulliparity, gestational age, maternal chronic conditions (for example, diabetes and hypertension), and the use of obstetric procedures (for example, continuous fetal monitoring and induction).

The birth registry dataset included six of the largest AA subgroups in the US (Asian Indian, Chinese, Filipino, Japanese, Korean, and Vietnamese) and one combined Other Asian subgroup which represented all other less populated AA subgroups including Hmong, Indonesian, Laotian, Pakistani, Sri Lankan, and Thai.

### 2.3. Data Analysis

Descriptive statistics were used to present the sociodemographic characteristics of participants in all racial and ethnic groups including AA subgroups. Chi-square analysis was used to compare the prevalence of PCD among racial and ethnic groups and among AA subgroups. Multivariable logistic regression models were performed to compare the prevalence of PCD between the AA group as a whole and other racial/ethnic groups, and within each of the AA subgroups controlling for sociodemographic and other known risk factors for the use of PCD. Additional multivariable logistic regression models were performed to compare the prevalence in each AA subgroup with NH-White women controlling for sociodemographic and other covariates. The clustering effect at the hospital level was controlled in the regression models. Missing data were treated as missing without data imputation due to our key variables having very little missing data. Data analysis was conducted using SAS^®^ Software version 9.4 (SAS Institute, Cary, NC, USA).

## 3. Results

### 3.1. Sample Characteristics

**Figure 1:**
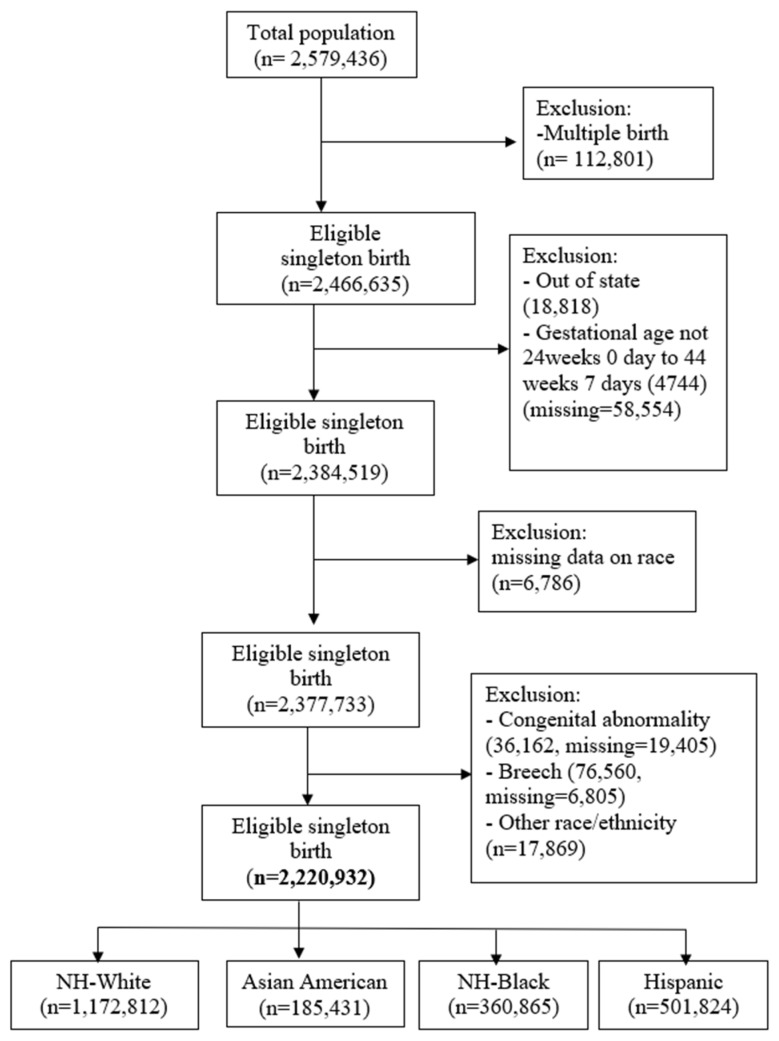
Flowchart for sample selection. Note: details of congenital anomalies attached as supplementary material S2.

There were 2,579,436 live births from 1992 to 2014 in our study population. After excluding multiple births, out-of-state births, births outside the timeframe of 24 to 44 gestational weeks, and records with missing data on the mothers’ race and gestational age, infant congenital abnormality and breech, the final analytic sample was 2,220,932 (Figure 1).

**Figure 2:**
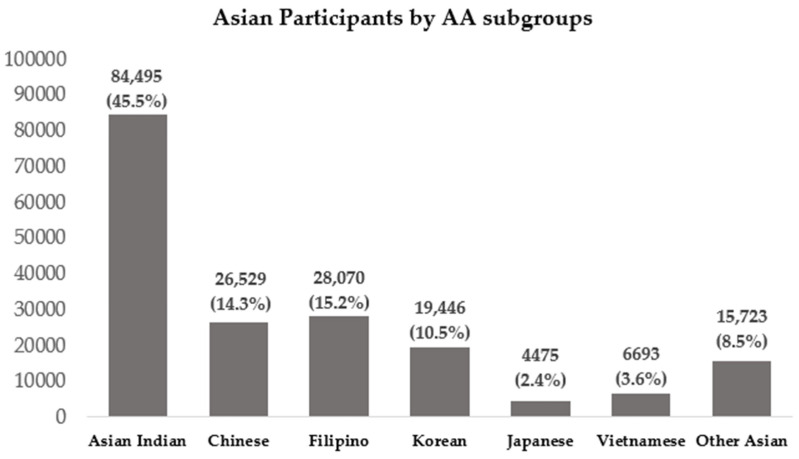
Asian participants by disaggregated Asian subgroups. (*n* = 185,431).

Most births were to NH-White women (*n* = 1,172,812, 52.8%), followed by Hispanic women (*n* = 501,824, 22.6%), NH-Black women (*n* = 360,865, 16.2%), and AA women (*n* = 185,431, 8.4%) (Table 1). The AA group was further divided into seven subgroups: Asian Indian (*n* = 84,495, 45.5%), Chinese (*n* = 26,529, 14.3%), Japanese (*n* = 4475, 2.4%), Korean (*n* = 19,446, 10.5%), and Vietnamese (*n* = 6693, 3.6%) (Figure 2).

### 3.2. AA Compared to All Other Racial and Ethic Groups

**Figure 3:**
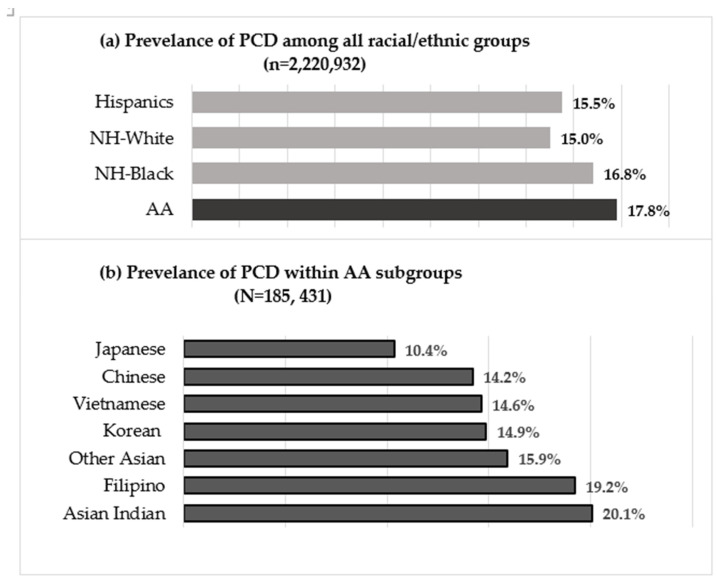
Prevalence of PCD, 1992–2014.

Compared to all other racial and ethnic groups, the AA women in this study were more likely to be nulliparous (first-time mothers) and married (*p* < 0.0001) (Table 1). In addition, they had the highest rate of diabetes (9.4%) and the lowest rate of hypertension (1.9%) compared to other women in the study (Table 1). However, infants born to AA women were more likely to be of normal weight (89.1%) and less likely to weigh more than 4000 g (4.7%) compared to other infants (Table 1). Yet, AA women had a higher PCD rate (17.8%) than any other racial and ethnic group (*p* < 0.0001) (Figure 3a).

### 3.3. Outcome within AA Subgroups

Within the AA subgroups, clear variations were observed in the rate of PCD use. The PCD prevalence was lowest among Japanese women (10.4%) and highest among Asian Indian women (20.1%) followed by Filipino (19.2%) with Chinese, Korean, Vietnamese, and other Asian subgroups in the middle range (14–16%) (Figure 3b).

After controlling for potential covariates, including maternal age, education, marital status, alcohol and tobacco use, the infant’s gender and birthweight, gestational age, nulliparity, diabetes, hypertension, initiation of prenatal care, use of continuous fetal monitoring, and induction, AA women were 19% more likely than NH-White women to have a PCD (Adj OR 1.19, 95% CI 1.17–1.21) but were less likely than NH-Black women (Adj. OR 0.78, 95% CI 0.77–0.80) and Hispanic women (Adj. OR 0.87, 95% CI 0.85–0.88) to have a PCD (Table 2).

**Figure 4:**
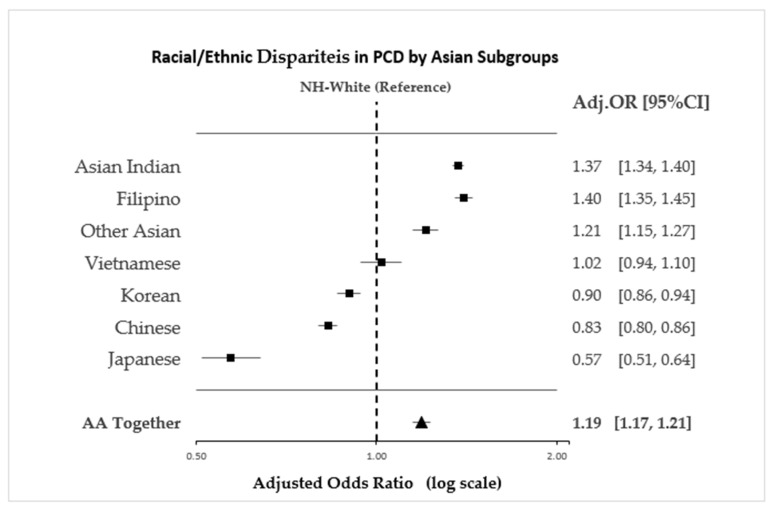
Racial/ethnic disparities in use of PCD among Asian Americans by disaggregated Asian subgroups, among women who had a singleton vertex live birth between 24 and 44 gestational weeks in a Northeastern state, 1992–2014. (*n* = 1,358,243). Model is adjusted for maternal age, education, marital status, alcohol and tobacco use, newborn’s gender, birth weight, gestational age, nulliparity, diabetes, hypertension, prenatal care, use of continuous fetal monitoring, and induction. Note: Other Asian includes Pakistani, Indonesian, Thai, Sri Lankan, Indonesian, and Hmong and Laotian women.

Extensive heterogeneity in the occurrence of PCD was found among the AA subgroups. After controlling for all covariates in the full model, Asian Indian (Adj. OR 1.37, 95% CI 1.34–1.40), Filipino (Adj. OR 1.40, 95% CI 1.35–1.45), and the combined Other Asian group of women (Adj. OR 1.21, 95% CI 1.15–1.27) had a higher risk for PCD compared to NH-White women. However, Chinese (Adj. OR 0.83, 95% CI 0.80–0.86), Japanese (Adj. OR 0.57, 95% CI 0.51–0.64), and Korean women (Adj. OR 0.90, 95% CI 0.86–0.94) had a lower risk of PCD compared to NH-White women, while the risk among Vietnamese women (Adj. OR 1.02, 95% CI 0.94–1.10) was not different from that of NH-White women (Figure 4).

## 4. Discussion

Among the women in this study, AAs had the highest PCD rate compared to any other racial and ethnic group and were more likely to have a PCD compared to NH-White women after controlling for socioeconomic characteristics, medical conditions, and behavioral and other risk factors. Moreover, significant variations were present among the different AA subgroups in their risk for PCD, with Asian Indian women presenting the highest risk (37% more likely to have a PCD than NH-White women), while Japanese were shown to have the lowest risk (43% less likely to have a PCD than NH-White).

Very few studies have examined racial and ethnic disparities in PCD prevalence or analyzed its use in AA women [12,13,22,23] or within AA subgroups [14]. However, our findings are consistent with those of Edmonds et al. [12] and Williams et al. [13] in that AA women, as a group, were at significantly increased risk of having an unplanned PCD compared to NH-White women. In addition, our findings are similar to those of another study carried out by Edmonds and colleagues [14], in that we also found that there are distinct heterogeneities among AA subgroups. As such, in our study, Asian Indian, Filipino, and an aggregated AA group comprising Hmong, Laotian, Indonesian, Pakistani, Sri Lankan, and Thai women are more likely to have a PCD and that Chinese, Japanese, and Korean women are less likely to have a PCD compared to NH-White women. Vietnamese women’s risk was found to be similar to NH-White women’s risk for PCD.

In a study that examined health but not birth outcomes, Adia et al. [24] highlighted that among AA subgroups, Filipino and Vietnamese persons were more likely to have fair or poor health compared to NH-Whites and other AA subgroups of women. As such, we must view the AA community as heterogenous. Recognizing that there are differences in birth and health outcomes within the larger AA population underscores the importance of examining AA subgroups so that patient education programs can be tailored to specific AA subgroups within the larger AA community.

### 4.1. Policy Implication

The persistent health disparities in maternal mortality present in the US are a public health priority [25], yet few studies have included AA participants without grouping them into an ‘other race’ category [11,12,26,27]. This approach has overlooked health disparities that may be present among AA women. From a public health perspective, this is of concern as racial disparities for AAs are prominent for some diseases. For example, AAs are more likely to have diabetes [28] which was also found in our study, and death rates due to Hepatitis B are more than eight times higher for AAs than for NH-Whites [29]. Additionally, infant mortality due to maternal health complications is 40% higher for AA women than for NH-White women [30]. These factors, together with the findings from our study, underscore the urgency and significance of addressing health disparities in AA women regarding maternal health outcomes, particularly as it relates to PCD. Our findings also underscore the importance of partnering with Asian American communities and organizations to better understand the diverse needs of AA subgroups in our local community so that, together, we can advance health equity.

### 4.2. Limitations

This study has some limitations. First, the birth registry data has been questioned for data quality but has been widely used in maternal and neonatal research because it is a unique population-level data resource. In this study, covariates included in our adjusted models might have been underreported in the state birth registry [31]. This could have potentially introduced a bias to our findings. In particular, if underreporting of congenital anomalies was present in the state birth registry data that we have used [32], it is possible that our sample did not exclude all congenital anomalies from the analysis. Second, not all the variables that we would like to have included in our analysis are collected in birth registry data. For example, both maternal obesity and diabetes can lead to fetal macrosomia, which increases the risk of CD [33,34]. Although we controlled for diabetes in our analysis, we were unable to adjust for maternal obesity as BMI was not available in our birth registry dataset. Another variable that is not included in birth registry data is the underlying reason for PCD, for example, was the PCD elective (planned) or due to medical reasons once birth began. Elective cesareans after 38 weeks are recommended by some in the medical community to prevent fetal macrosomia [34] and some medical centers might have a higher usage of elective PCD than others due to hospital expediency and guidelines [11] Although information related to elective PCD was not available in our dataset, each hospital or birth center that the study participant gave birth at had a unique institutional code. Therefore, in the multivariable regression models, we were able to control for the cluster effect at the hospital level. This maneuver should have alleviated the impact on our study findings caused by institutional related elective PCD use. Additionally, birth record data do not inform researchers about whether the AA woman was born in the US or outside of the US, and this may impact the study findings.

## 5. Conclusions

The racial and ethnic health disparities among AA women in relation to the high prevalence of PCD use warrant attention. Overt variation in the PCD rate was observed within AA subgroups, with Asian Indian and Filipino women experiencing the highest burden. Viewing all AA subgroups as a single entity under the larger umbrellas of “AA” prevents healthcare providers and policy makers from correctly identifying the needs and necessary interventions to address health disparities within the larger entity of AA. More studies focusing on maternal health of AA populations, especially those with desegregated AA subgroups, are warranted for evidence-based health care and policy making.

## Figures and Tables

**Table 1:** Sociodemographic and other risk factors for PCD by race and ethnicity groups among women who had a singleton vertex live birth, with gestational age between 24 weeks and 44 weeks, in a Northeastern State, 1992–2014 (N = 2,220,932).

	AA *n* (%)	NH-White *n* (%)	NH-Black *n* (%)	Hispanic *n* (%)	*p*-Value *
Total N	185,431 (8.4)	1,172,812 (52.8)	360,865 (16.2)	501,824 (22.6)	
Sociodemographic					
Age Groups					
≤20	2484 (1.3)	53,510 (4.6)	76,348 (21.2)	84,937 (16.9)	<0.0001
21–34	144,282 (77.8)	846,708 (72.2)	238,174 (66.0)	353,892 (70.5)	
≥35	38,651 (20.9)	272,502 (23.2)	46,274 (12.8)	62,961 (12.6)	
Education					
Less than HS	6885 (3.8)	58,214 (5.0)	68,122 (19.4)	169,920 (34.5)	<0.0001
HS completed	21,015 (11.5)	313,009 (27.0)	143,497 (40.8)	175,719 (35.7)	
Attended Coll.	154,867 (84.7)	787,935 (68.0)	139,776 (39.8)	147,222 (29.9)	
Married					<0.0001
Yes	176,018 (94.9)	999,612 (85.2)	115,868 (32.1)	224,268 (44.7)	
Newborn birth weight					
<2500 g	12,304 (6.3)	50,693 (4.1)	42,057 (10.9)	30,452 (5.8)	<0.0001
2500–3999 g	175,086 (89.1)	1,040,545 (83.5)	320,522 (83.2)	457,614 (86.4)	
≥4000 g	9178 (4.7)	154,652 (12.4)	22,684 (5.9)	41,753 (7.9)	
Maternal Characteristics					
Diabetes Mellitus	18,474 (9.43)	47,263 (3.81)	16,740 (4.39)	27,418 (5.20)	<0.0001
Hypertension	3688 (1.88)	34,287 (2.77)	14,350 (3.76)	12,477 (2.37)	<0.0001
Prenatal Care					
No Care	558 (0.23)	5776 (0.5)	14,117 (3.8)	6084 (1.2)	
Care start T1	165,470 (85.5)	1,084,794 (88.8)	240,423 (64.5)	363,648 (69.8)	
Care start T2	22,300 (11.5)	109,093 (8.9)	93,124 (25.0)	125,160 (24.0)	<0.0001
Care start T3	5124 (2.7)	22,169 (1.8)	24,843 (6.7)	26,117 (5.0)	
Nulliparous	95,126 (48.44)	523,644 (42.12)	153,761 (40.01)	209,162 (39.53)	<0.0001

Note. HS = high school, AA = Asian American, T1, T2, T3 = Trimester 1, Trimester 2, Trimester 3, PCD = primary cesarean delivery. * *p*-value: Chi-square test.

**Table 2:** Racial disparities associated with the use of PCD among women who had a singleton vertex live birth between 24 and 44 gestational weeks in a Northeastern state, 1992–2014. (*n* = 2,220,932).

	Using PCD	
	Raw Model OR (95% CI)	Adjusted Model * AOR (95%CI)
AA vs. NH-White	1.22 (1.21–1.24)	1.19 (1.17–1.21)
AA vs. NH-Black	1.06 (1.05–1.08)	0.78 (0.77–0.80)
AA vs. Hispanic	1.18 (1.16–1.19)	0.87 (0.85–0.88)

Note. NH = Non-Hispanic. Cluster effect at hospital level was controlled. * Model adjusted for maternal age, education, marital status, alcohol and tobacco use, newborn’s gender, birth weight, gestational age, nulliparity, diabetes, hypertension, prenatal care, use of continuous fetal monitoring, and induction.

## Data Availability

Data presented in this study are birth registry data publicly available upon request from the Department of Health at the state level.

## Supplementary Materials

The following supporting information can be downloaded at: https://www.mdpi.com/article/10.3390/ijerph20196860/s1, Figure S1: PCD trends across racial groups, 1992–2014. Form S2: Congenital Anomalies of the Child.
