# Supplementary material for: Health Disparities in the Use of Primary Cesarean Delivery among Asian American Women
Source: Int J Environ Res Public Health. 2023 Sep 29;20(19):6860. doi: 10.3390/ijerph20196860 (PMC10572660; doi:10.3390/ijerph20196860)
Supplement: Supplementary file 1 [file ijerph-20-06860-s001.zip › ijerph-2432372-supplementary.pdf]

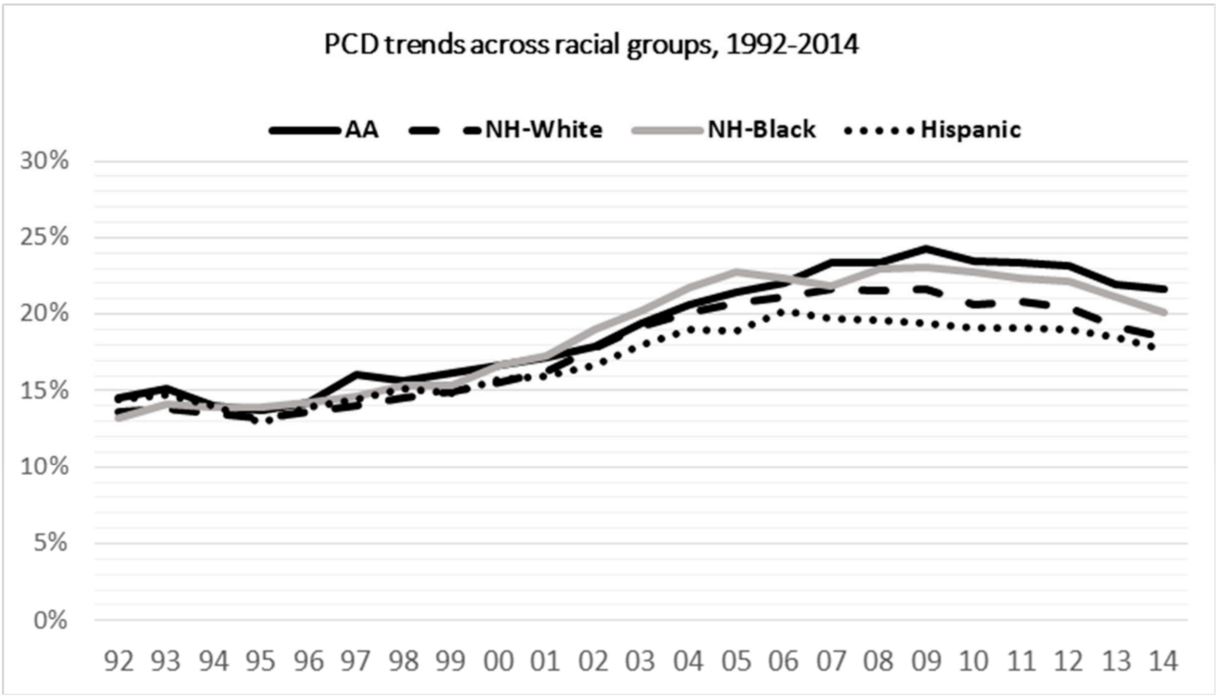

Figure S1: PCD trends across racial groups, 1992-2014.

Supplementary S2: CONGENITAL ANOMALIES OF THE CHILD

FIELD NUMBER & DESCRIPTION:
99 – CONGENITAL 100 – OTHERCNS 101 – OTHERCIRC
102 – OTHERGAST 103 – OTHERURO 104 – OTHERMUSC
105 – OTHERCHROM 106 – OTHERCONGEN
DEFINITION: Birth defects, as specified by the National Center for Health Statistics, which are known at the time the birth certificate is issued. Clinical judgment must be used in the determination of anomalies.
REQUIRED FOR JCAHO: NO
FIELD LENGTH & TYPE: 23/NUMERIC
56/ALPHA
MANDATORY: YES
CAN BE UNKNOWN: YES
ACCEPTABLE ENTRY: Select all choices that apply from the pop-up window.
Do not include birth injuries.

CENTRAL NERVOUS SYSTEM
01 [ ] Anencephalous
02 [ ] Spina bifida/Meningocele
03 [ ] Hydrocephalus
04 [ ] Microcephalus
05 [ ] Other Central Nervous System
Anomalies, specify ________

HEART
06 [ ] Heart Malformations
07 [ ] Other Circulatory/Respiratory
Anomalies, specify ________

GASTROINTESTINAL
08 [ ] Rectal Atresia/Stenosis
09 [ ] Tracheoesophageal Fistula/
Esophageal Atresia
10 [ ] Omphalocele/Gastroschisis
11 [ ] Other Gastrointestinal
Anomalies, specify ________

UROGENITAL
12 [ ] Malformed Genitalia
13 [ ] Renal Agenesis
14 [ ] Other Urogenital
Anomalies, specify ________

MUSCULOSKELETAL
15 [ ] Cleft Lip/Palate
16 [ ] Polydactyly/Syndactyly/Adactyly
17 [ ] Club Foot
18 [ ] Diaphragmatic Hernia
19 [ ] Other Musculoskeletal/Integumental

Anomalies, specify ________

CHROMOSOMAL
20 [ ] Down's Syndrome
21 [ ] Other Chromosomal
Anomalies, specify ________
22 [ ] Other, Not Covered Elsewhere
23 [ ] Unknown-Diagnostic Tests
Ordered or Diagnosis Tests
Possible
00 [ ] None
